# Is Health Literacy of Dialyzed Patients Related to Their Adherence to Dietary and Fluid Intake Recommendations?

**DOI:** 10.3390/ijerph16214295

**Published:** 2019-11-05

**Authors:** Ivana Skoumalova, Peter Kolarcik, Andrea Madarasova Geckova, Jaroslav Rosenberger, Maria Majernikova, Daniel Klein, Jitse P. van Dijk, Sijmen A. Reijneveld

**Affiliations:** 1 Department of Health Psychology, Faculty of Medicine, PJ Safarik University, Trieda SNP 1, 040 11 Kosice, Slovakia; peter.kolarcik@upjs.sk (P.K.); andrea.geckova@upjs.sk (A.M.G.); jaroslav.rosenberger@upjs.sk (J.R.); 2 Graduate School Kosice Institute for Society and Health, PJ Safarik University, Trieda SNP 1, 040 11 Kosice, Slovakia; j.p.van.dijk@umcg.nl; 3 Department of Community & Occupational Health, University Medical Center Groningen, University of Groningen, Antonius Deusinglaan 1, 9713 AV Groningen, The Netherlands; s.a.reijneveld@umcg.nl; 4 Olomouc University Social Health Institute, Palacky University in Olomouc, Univerzitni 22, Olomouc 771 11, Czech Republic; 5 II. Internal Clinic, Faculty of Medicine, P.J. Safarik University, Trieda SNP 1, 040 11 Kosice, Slovakia; 6 FMC-Dialysis Services Slovakia, Trieda SNP 1, 040 11 Kosice, Slovakia; mamajern@gmail.com; 7 Institute of Mathematics, Faculty of Science, P. J. Safarik University, Jesenna 5, 040 01 Kosice, Slovakia; daniel.klein@upjs.sk

**Keywords:** health literacy, diet adherence, fluid intake adherence, dialyzed patients, non-adherence

## Abstract

Non-adherence to dietary and fluid intake recommendations (NADFIR) is an important factor for the effective treatment of dialyzed patients and may be hindered by low health literacy (HL). Therefore, we assessed whether low HL of dialyzed patients is associated with their NADFIR. We performed a multicentric cross-sectional study in 20 dialysis clinics in Slovakia (*n* = 452; response rate: 70.1%; mean age = 63.6 years; males: 60.7%). We assessed the association between nine domains of HL and non-adherence (high serum potassium, high serum phosphate, relative overhydration, and self-reported NADFIR) using general linear models adjusted for age and gender. Moreover, we assessed the moderation by socioeconomic status (SES). We found higher NADFIR among patients with less sufficient information for health management (high serum phosphate level; odds ratio (OR): 0.77; 95% confidence interval (CI): 0.63–0.94), with a lower ability to actively manage their health (self-reported diet non-adherence; OR: 0.74; 95% CI: 0.62–0.89), and those less able to actively engage with healthcare providers (overhydrated; OR: 0.78; 95% CI: 0.65–0.94). Moreover, SES modified this relation. Low HL affects the adherence of dialyzed patients. This shows a need to support patients with low HL and to train healthcare providers to work with these patients, taking into account their SES.

## 1. Introduction

Chronic kidney disease (CKD) is one of the major public health problems worldwide, with a prevalence of 8%–16% and representing a great economic burden [1,2,3,4]. Its prevalence is constantly rising as a result of increasing rates of risk factors, such as diabetes mellitus, hypertension, and obesity. In 2015, CKD was the 12th most frequent cause of death [5]. The disease has the tendency to progress to end-stage kidney disease in older age [1,3], affecting approximately 2% of the total CKD population [6]. To maintain the life of a patient in this stage of the disease, hemodialysis therapy or transplantation is inevitable.

In CKD, adherence to dietary and fluid intake recommendations as a part of self-management is very important for an effective treatment that reduces complications, improves the overall quality of life, and decreases mortality [7,8]. This holds even more so for its advanced stages. Adherence can be defined as the extent to which the patient’s behavior corresponds with recommendations from a healthcare provider [9]. Dialyzed patients have to monitor their fluid, sodium, potassium, and phosphate intakes, making their diet very restrictive. Previous research on adherence to dietary and fluid intake recommendations in dialyzed patients shows an adherence to being poor with a mean of 31.5% for dietary recommendations and 68.5% for fluid intake recommendations [10,11]. Research further shows that poor adherence is associated with an increased risk of death and hospitalization [12,13,14,15]. Dialyzed patients report that the major reasons for poor adherence are that the diet is very complicated, frustrating, and demanding to manage [16,17,18]. Many factors may influence adherence, such as the length and severity of illness as well as comorbidities, self-efficacy, social support, depression, and anxiety [19], including health literacy (HL) [20].

Poor HL is likely to lead to poor adherence, but evidence on this is limited. HL is defined by Sørensen et al. [21] as being linked to literacy and entails people’s knowledge, motivation, and competence to access, understand, appraise, and apply health information in order to make judgments and decisions in their everyday life concerning healthcare, disease prevention, and health promotion to maintain or improve their quality of life. Limited HL is common in dialyzed patients [22,23] and has been shown to be associated with missed dialysis sessions, higher rates of hospitalization, higher mortality rates [24,25], and reduced access to kidney transplantation [26]. Lower socioeconomic status (SES) is associated with limited HL according to previous research [22,27], as well as with non-adherence to dietary and fluid intake recommendations (NADFIR) [10].

Previous research has focused mainly on the attendance of dialysis sessions and adherence to prescribed medication in association with HL, showing a lower adherence in patients with lower HL [28,29]. However, the evidence on adherence to dietary and fluid intake recommendations in association with patients’ HL, specifically in dialyzed patients, is still lacking. Although we know about the disadvantageous effect of lower SES on patient adherence, its role in the association between HL and adherence in dialyzed patients has not been sufficiently explained. We hypothesize that patients with lower health literacy will be more likely to be non-adherent to dietary and fluid intake recommendations and that SES will moderate this relation. Therefore, our aim was to assess the associations of multidimensional HL with non-adherence to dietary and fluid intake recommendations in dialyzed patients, controlled for patients’ gender, age, and sociodemographic characteristics. Moreover, we assessed whether SES moderated the association of HL with non-adherence.

## 2. Materials and Methods

### 2.1. Sample and Procedure

We collected data from January to November 2018 within a network of 20 dialysis clinics belonging to one private network (FMC-Dialysis services Slovakia) covering approximately 20% of the total Slovak dialysis population. This dialysis therapy is fully reimbursed by a compulsory health insurance system. The inclusion criteria required patients to be over the age of 18, have a diagnosis of stage 5 CKD, and have been undergoing hemodialysis treatment for at least 90 days. The exclusion criteria consisted of an inability to fill in the questionnaire (due to dementia, mental retardation, or an inability to read the Slovak language), acute severe intercurrent illness, and the presence of a psychiatric diagnosis, which similar to other studies focusing on dialyzed patients [11,23,24,28].

The data were obtained by questionnaires filled in by patients during their routine visits to the dialysis clinic and by extracting from medical records. Patients agreed to participate in the study by signing an informed consent form prior to the study. Then, they filled in questionnaires using tablets with data recorded to an online platform and full confidentiality assured with a personal identification code. Regarding the latter, we included those recordings closest to the completion of the questionnaires. A research assistant was available for technical support. The clinical data were obtained from the European Clinical Database (EuCliD5), in which all medical record data from the Fresenius clinics are stored.

### 2.2. Ethics

The study was approved by the Ethics Committee of the Faculty of Medicine, Pavol Jozef Safarik University (15N/2017), and the Ethics Committee of FMC-Dialysis services (23 November 2017). All the data and information from the documentation, including demographic and clinical data, were used in accordance with the ethical standards as laid out in the 1964 Declaration of Helsinki and its later amendments or comparable ethical standards.

### 2.3. Measures

Non-adherence was measured based on clinical measures and patient self-reports. The clinical measures of non-adherence to diet and fluid intake guidelines regarded relative overhydration (≥13% (female), ≥15% (male), a high serum phosphate level (≥1.78 mmol/L), and a high serum potassium level (>5.5 mmol/L). These were obtained from the medical database (EuCliD5) [30] and dichotomized. They are the key performance indicators in the dialysis clinics where the study was conducted and based on international renal guidelines [31].

Self-reported non-adherence to diet and fluid guidelines was measured with a questionnaire on the frequency of breaking dietary and fluid intake recommendations separately, with six answers offered: (1) do not break; (2) once a month or less; (3) once per 2–3 weeks; (4) once a week; (5) 2–3 times a week; (6) always. The respondents were considered non-adherent if they broke the recommendations at least once a week (cutoff point = 3) with respect to the disease-specific guidelines [31] and opinions of renal experts, and following the approaches of previous studies regarding adherence where available [10].

Health literacy was measured using the Slovak version of the Health Literacy Questionnaire (HLQ) [32], a multidimensional tool for measuring nine domains of HL [33]. The questionnaire consisted of nine highly reliable domains of HL (composite reliability indices ranging from 0.73 to 0.84 for each of the 4- to 6-item subscales [31]) related to accessing, understanding, and using information to make decisions about health (see Appendix A for measurement tool information). Higher mean scores in a particular domain indicated a better HL. There was no overall total score for this measurement tool. For further analyses of HL, the expectation maximization algorithm [34] was used to replace missing HLQ scores per domain for each respondent at a maximum of two missing values for the 4–5 items/domains and at a maximum of three missing values from the 6-item domain [35]. We excluded respondents who had more missing scores in a particular domain.

Sociodemographic data were measured using the questionnaire and included age, gender, and SES. SES was measured with the MacArthur Scale of Subjective Social Status [36], a single-item measure that assesses a respondent’s perceived rank relative to others. The respondents were asked to rate their subjective social status on a drawing of a ladder with the instruction: “At the top of the ladder are the people who are the best off, those who have the most money, most education, and best jobs. At the bottom are the people who are the worst off, those who have the least money, least education, worst jobs, or no job. Please mark the number that best represents where you think you stand on the ladder.” The obtained scores ranged from 0 (the worst SES) to 10 (the best SES) and were used as a continuous variable. This instrument has been used in previous studies investigating older patients and patients with various health conditions [37,38].

### 2.4. Statistical Analyses

First, we assessed the sociodemographic characteristics, dietary, and fluid non-adherence of the sample. Second, we assessed the associations of the continuous-level scores on the nine domains of HL with the dichotomized clinical and self-reported indicators of non-adherence, using binary logistic regression in generalized linear models (GLM). We adjusted these analyses for age (continuous-level variable), gender, and SES (continuous-level variable). All continuous-level variables were used as standardized, i.e., *z*-scores. For each outcome, we reported an odds ratio (OR) with 95% confidence interval (CI), and *p* < 0.01 was assumed for statistical significance to adjust for multiple testing [39].

Finally, we tested the moderating effect of SES on the association of HL and non-adherence. All statistical analyses were performed using SPSS v. 23.0 for Windows [40].

## 3. Results

### 3.1. Baseline Characteristics

We included 567 patients on maintenance dialysis (70.1% of those approached), and 25 of these were excluded due to not filling in the questionnaire related to HL (*n* = 9) or missing too many items in it (*n* = 16). This led to a final sample of 542 patients (Table 1). The prevalence of non-adherence in our sample ranged from 21.7% to 43.3% for specific clinical and self-reported indicators. The patients’ self-report of non-adherence led to higher estimates than the clinical indicators. The sample mainly consisted of older people, and mostly men.

### 3.2. Associations of Health Literacy per Domain and Non-Adherence

We found higher a non-adherence for dietary and fluid recommendations among patients with lower HL (see Table 2). The patients with less sufficient information for managing health (HLQ2) were more likely to have high serum phosphate levels (OR: 0.77; 95% CI: 0.63–0.94). Those with a lower ability to actively manage their health (HLQ3) were more likely to report non-adherence to diet recommendations (OR: 0.74; 95% CI: 0.62–0.89), and those less able to actively engage with healthcare providers (HLQ6) were more likely to be overhydrated (OR: 0.78; 95% CI: 0.65–0.94).

### 3.3. Moderating Effect of SES on the Association between HL Domains and Non-Adherence

In Table 2, we also presented the moderating effect of SES on the association of HL domains with non-adherence. We found that SES moderated the association of the ability to actively engage with healthcare providers (HLQ6), navigate the healthcare system (HLQ7), and understand health information well enough to know what to do (HLQ9) with serum potassium non-adherence. The interaction indicates that patients with lower SES were more likely to be non-adherent, i.e., to have a high serum potassium level than patients with higher SES in the case of lower HL in these three domains of HL.

We also found that SES moderated the association of the ability to actively engage with healthcare providers (HLQ6) with self-rated fluid non-adherence. The patients with lower SES were less likely to report non-adherence with fluid intake recommendations than those with higher SES in the case of lower HL, e.g., a lower ability to engage with healthcare providers.

## 4. Discussion

In our study, we explored the association of the nine domains of HL with dietary and fluid intake non-adherence in dialyzed patients in Slovakia, and the moderation effect of SES on this relation. We found that not having sufficient information to manage health, being less active in managing health, and being less able to actively engage with healthcare providers were associated with non-adherence to dietary and fluid intake recommendations. Moreover, SES was shown to be a significant moderator in this relation.

We found an association of lower HL with non-adherence to both diet and fluid intake. We cannot compare this to previous findings as evidence on adherence to dietary and fluid intake recommendations in association with patients’ HL in dialyzed patients lacked until now. Despite the fact that adherence to dietary and fluid intake recommendations is an important factor for the effective treatment of dialyzed patients, limited HL is common in this patient group [22,23] and showed to be associated with their non-adherence [28,29]. A recent review [10] on diet adherence in dialyzed patients did not identify any research specifically focusing on patients’ HL. Nevertheless, there are at least two studies [41,42] exploring the association of HL with serum phosphate level as an indicator of non-adherence to diet. Dodson et al. [42] used the same measurement of HL (HLQ), but with a smaller studied sample (*n* = 100) and with participants receiving three different types of dialysis, and found no difference in serum phosphate levels when comparing patients with higher and lower HL. Jain et al. [41] measured functional HL and found no association in this relation. Moreover, they reported a trend toward higher serum phosphate in patients with higher functional HL. Thus, low HL indeed seems to hamper patients’ understanding of adherence to various guidelines that are essential for adequate CKD treatment.

Our research showed that SES moderated the association between HL and adherence to diet and fluid intake, but our findings are inconsistent. In cases of adherence to diet (high serum potassium level), we found patients with lower SES to be more vulnerable, but in cases of adherence to fluid intake (self-rated fluid non-adherence), we found them to be less vulnerable than patients with higher SES. The findings of previous research on the association between SES and adherence are inconsistent [10]. The inconsistent role of SES in the association of HL and non-adherence could be due to several factors in this patient group. Dialyzed patients have to involve various individual skills and also capacities in communication to be able to adhere to the complex requirements of their treatment regarding diet and fluid intake. Moreover, their success with adherence requires certain conditions, i.e., not only their individual capacity to adhere but also their socioeconomic background, which promotes or hinders adherence to specific fluid and diet guidelines. Stomer et al. [43] found that a lower level of education was associated with lower scores in the “Ability to navigate the healthcare system”, “Find good health information”, and “Understand health information well enough to know what to do” scales, in a sample consisting of CKD patients (stages 3–5). Lambert, Mulan, and Mansfield [10] also found that patients with lower education showed worse adherence. In addition, younger adults and those who were working showed worse dietary adherence. This might be explained by the fact that younger and working patients lack the capacity for better self-management behavior related to diet, or they do not have the conditions appropriate to perform it, potentially also affecting the role of SES in this association. In summary, the effect of HL on adherence may depend on a number of factors associated with SES, with the mechanisms requiring further study.

### 4.1. Strengths and Limitations

The major strength of our study was the representativeness of our sample, enabling our findings to be generalized to the population of dialyzed patients, its focus on multidimensional HL, and on adherence to diet and fluid intake, using a wide range of measures. Capturing the multidimensional and contextual nature of HL [21,44] offered a more comprehensive insight into HL needs and limitations related to the capacity to adhere with dietary and fluid intake recommendations [35,45] in dialyzed patients. Most research on HL in dialyzed patients has been based on one-dimensional tools to assess HL [20,25], mostly measuring functional HL, i.e., basic reading and writing skills related to health information and health services utilization [46]. Recent and more critical approaches to address HL consider these one-dimensional tools as insufficient [47,48].

The major limitation of this study regards the multiple testing that increased the probability of false positives. To avoid this, we used a Bonferroni correction and focused only on statistical significance at *p* < 0.01. Another limitation might be the cross-sectional design of our study, as this type of design did not allow us to describe any causal pathways between the studied variables.

### 4.2. Implications

Our finding that low HL is associated with poorer adherence shows a need to increase the responsiveness of the healthcare system to various patients’ HL needs and limitations, i.e., to support patients who are less likely to be active in communicating about their symptoms and concerns related to their health condition, or about problems with adherence to treatment recommendations. This might include providing them with information about proper adherence, so they can be sure that they are able to meet them, and to emphasize and support patients’ activity in self-management along with shared decision-making about their health. The clinical relevance of our findings lies in the association of non-adherence with increased morbidity and mortality. The adverse health outcomes might be reversed with interventions focused on increasing HL capacities on the side of the patient as well as on that of the healthcare system. Regular screening of HL in dialyzed patients should inform healthcare providers about their HL needs and limitations and, thus, foster more effective support of patients’ adherence to dietary and fluid intake recommendations.

Future research should also include patients in earlier stages of the disease as well as on other dialysis modalities in order to explore the differences and variability in the HL domains in the context of non-adherence to dietary and fluid intake recommendations. This may also allow the exploration of various social, economic, and educational backgrounds. It should also inform on mediating and moderating factors in this relation and should therefore clarify the role of HL in the context of non-adherence of dialyzed patients within other factors entering into this mechanism [49].

## 5. Conclusions

We found that not having sufficient information for managing health, being less active in managing health, and being less able to actively engage with healthcare providers was associated with non-adherence to dietary and fluid intake recommendations. Moreover, SES showed up as a significant moderator in this relation. These findings point to the need to support patients with varying HL limitations and to further develop the capacity of healthcare providers to work with patients with low HL while considering their socioeconomic background.

## Figures and Tables

**Table 1:** Characteristics of the sample: gender, age, and socioeconomic status (SES); measures of non-adherence and health literacy (HL); frequencies or means (*n* = 542 patients from 20 dialysis clinics in Slovakia interviewed in 2018).

Sociodemographic Data	*n*	%	M	SD
Male gender	329	60.7		
Age			63.58	14.12
Socioeconomic status ^1^			5.16	1.88
Non-adherence				
High serum phosphate level	152	28.4		
High serum potassium level	116	21.7		
Relative overhydration	169	32.3		
Diet non-adherence	229	43.3		
Fluid intake non-adherence	203	38.4		
Health literacy				
HLQ1—Feeling understood and supported by healthcare provider ^2^			3.22	0.46
HLQ2—Having sufficient information to manage health ^2^			3.12	0.46
HLQ3—Actively managing my health ^2^			3.06	0.44
HLQ4—Social support for health ^2^			3.23	0.47
HLQ5—Appraisal of health information ^2^			2.90	0.53
HLQ6—Ability to actively engage with healthcare providers ^3^			3.90	0.67
HLQ7—Navigating the healthcare system ^3^			3.70	0.72
HLQ8—Ability to find good health information ^3^			3.77	0.70
HLQ9—Understand health information well enough to know what to do ^3^			3.80	0.65

^1^ The range of score is 0–10 (the lowest SES–the highest SES); ^2^ The range of mean score 1–4; ^3^ The range of mean score 1–5.

**Table 2:** The associations between health literacy domains and non-adherence, and the moderating effect of socioeconomic status on the association between health literacy and non-adherence (*n* = 542, patients from 20 dialysis clinics in Slovakia interviewed in 2018). General linear models adjusted for age and gender (odds ratio and 95% confidence intervals).

	High Serum Phosphate Level *n* = 533	High Serum potassium Level *n* = 530	Relative Overhydration *n* = 520	Self-Rated Diet Non-adherence *n* = 526	Self-Rated Fluid Non-adherence *n* = 526
Feeling understood and supported by health care provider (HLQ1)	0.83 (0.68–1.01)	1.13 (0.92–1.40)	0.85 (0.70–1.03)	0.89 (0.75–1.07)	0.99 (0.83–1.19)
Socio-economic status	0.91 (0.75–1.11)	1.04 (0.85–1.28)	1.23 (1.02–1.49)	1.06 (0.89–1.26)	0.97 (0.81–1.16)
HLQ1 ● Socio-economic status					
Having sufficient information to manage health (HLQ2)	0.77 (0.63–0.94) **	0.97 (0.79–1.20)	0.87 (0.72–1.05)	0.81 (0.67–0.97)	0.96 (0.80–1.15)
Socio-economic status	0.91 (0.75–1.10)	1.05 (0.85–1.29)	1.23 (1.02–1.49)	1.06 (0.89–1.27)	0.97 (0.81–1.17)
HLQ2 ● Socio-economic status					
Actively managing my health (HLQ3)	0.81 (0.66–0.99)	0.99 (0.80–1.21)	0.86 (0.71–1.04)	0.74 (0.62–0.89) **	0.79 (0.66–0.96)
Socio-economic status	0.92 (0.76–1.11)	1.05 (0.85–1.29)	1.24 (1.02–1.50)	1.07 (0.90–1.28)	0.99 (0.82–1.18)
HLQ3 ● Socio-economic status					
Social support for health (HLQ4)	0.92 (0.76–1.12)	1.16 (0.94–1.43)	0.86 (0.72–1.04)	0.90 (0.76–1.08)	0.96 (0.80–1.15)
Socio-economic status	0.91 (0.75–1.10)	1.04 (0.85–1.28)	1.23 (1.02–1.49)	1.06 (0.89–1.26)	0.97 (0.81–1.17)
HLQ4 ● Socio-economic status					
Appraisal of health information (HLQ5)	0.84 (0.68–1.02)	0.91 (0.74–1.12)	1.05 (0.87–1.27)	1.05 (0.88–1.26)	0.93 (0.77–1.12)
Socio-economic status	0.93 (0.76–1.12)	1.06 (0.86–1.31)	1.21 (1.00–1.47)	1.05 (0.88–1.25)	0.98 (0.82–1.18)
HLQ5 ● Socio-economic status					
Ability to actively engage with health care providers (HLQ6)	0.82 (0.67–0.99)	0.99 (0.80–1.23)	0.78 (0.65–0.94) **	0.96 (0.80–1.14)	1.07 (0.89–1.29)
Socio-economic status	0.90 (0.75–1.09)	1.07 (0.86–1.32)	1.22 (1.01–1.47)	1.05 (0.88–1.30)	0.96 (0.80–1.16)
HLQ6 ● Socio-economic status		0.73 (0.58 – 0.92) **			1.31 (1.07 – 1.59) ***
Navigating the health care system (HLQ7)	0.78 (0.64–0.95)	0.97 (0.78–1.20)	0.82 (0.68–0.99)	0.93 (0.78–1.11)	1.02 (0.85–1.22)
Socio-economic status	0.93 (0.76–1.12)	1.10 (0.89–1.37)	1.24 (1.03–1.51)	1.06 (0.89–1.26)	0.97 (0.81–1.16)
HLQ7 ● Socio-economic status		0.70 (0.54 – 0.90) **			
Ability to find good health information (HLQ8)	0.78 (0.64–0.95)	1.06 (0.85–1.31)	0.85 (0.70–1.03)	0.95 (0.79–1.14)	1.02 (0.84–1.22)
Socio-economic status	0.93 (0.77–1.14)	1.04 (0.85–1.28)	1.24 (1.03–1.50)	1.06 (0.89–1.26)	0.97 (0.81–1.16)
HLQ8 ● Socio-economic status					
Understand health information well enough to know what to do (HLQ9)	0.83 (0.68–1.01)	1.05 (0.84–1.31)	0.87 (0.72–1.05)	0.85 (0.71–1.01)	0.92 (0.76–1.10)
Socio-economic status	0.93 (0.77–1.12)	1.09 (0.88–1.35)	1.24 (1.03–1.51)	1.07 (0.90–1.28)	0.98 (0.82–1.18)
HLQ9 * Socio-economic status		0.70 (0.55–0.91) **			

** *p* < 0.01, *** *p* < 0.001; Cut-off points: High serum phosphate level: ≤1.78 mmol/l; High serum potassium level: <5.5 mmol/l; Relative overhydration: ≤13% (female), ≤15% (male); Self-rated diet and fluid non-adherence: at least once a week; Model with interaction between HLQ and SES on non-adherence is presented only in case when it was significant. Otherwise model without interaction is presented.

## Appendix A

### Health Literacy Questionnaire—Detailed Description of the Measurement Tool

The instrument is divided into two parts with different instructions and answering options. The mean score for each domain is used for analysis.

In the first part, which covers domains 1–5, respondents are asked to what extent they agree with the statements. Answering options include four response categories: 1—strongly agree; 2—agree; 3—disagree; 4—strongly disagree. Domains 1–5 have a mean score ranging from 1–4. These domains are: (1) Feeling understood and supported by healthcare provider; (2) Having sufficient information to manage my health; (3) Actively managing my health; (4) Social support for health; (5) Appraisal of health information.

In the second part, which covers domains 6–9, respondents are asked how easy or difficult certain tasks are for them. Answering options include five response categories: 1—Cannot do; 2—Very difficult; 3—Quite difficult; 4—Quite easy; 5—Very easy. Domains 6–9 have a mean score ranging from 1–5. These domains are (6) Ability to actively cooperate with healthcare providers; (7) Navigating the healthcare system; (8) Ability to find good health information; and (9) Understanding health information well enough to know what to do.

## References

[1] Coresh J., Astor B.C., Greene T., Eknoyan G., Levey A.S. (2003). Prevalence of chronic kidney disease and decreased kidney function in the adult US population: Third National Health and Nutrition Examination Survey. Am. J. Kidney Dis..

[2] Hill N.R., Fatoba S.T., Oke J.L., Hirst J.A., O’Callaghan C.A., Lasserson D.S., Hobbs F.D. (2016). Global Prevalence of Chronic Kidney Disease—A Systematic Review and Meta-Analysis. PLoS ONE.

[3] Jha V., Garcia-Garcia G., Iseki K., Li Z., Naicker S., Plattner B., Saran R., Wang A.Y., Yang C.W. (2013). Chronic kidney disease: Global dimension and perspectives. Lancet.

[4] Levey A.S., Atkins R., Coresh J., Cohen E.P., Collins A.J., Eckardt K.U., Nahas M.E., Jaber B.L., Jadoul M., Levin A. (2007). Chronic kidney disease as a global public health problem: Approaches and initiatives—A position statement from Kidney Disease Improving Global Outcomes. Kidney Int..

[5] GBD 2015 Mortality and Causes of Death Collaborators (2016). Global, regional, and national life expectancy, all-cause mortality, and cause-specific mortality for 249 causes of death, 1980–2015: A systematic analysis for the Global Burden of Disease Study 2015. Lancet.

[6] Anderson S., Halter J.B., Hazzard W.R., Himmelfarb J., Horne F.M., Kaysen G.A., Kusek J.W., Nayfield S.G., Schmader K., Tian Y. (2009). Prediction, progression, and outcomes of chronic kidney disease in older adults. J. Am. Soc. Nephrol..

[7] Ash S., Campbell K.L., Bogard J., Millichamp A. (2014). Nutrition prescription to achieve positive outcomes in chronic kidney disease: A systematic review. Nutrients.

[8] Kang S.S., Chang J.W., Park Y. (2017). Nutritional Status Predicts 10-Year Mortality in Patients with End-Stage Renal Disease on Hemodialysis. Nutrients.

[9] Rand C.S. (1993). Measuring adherence with therapy for chronic diseases: Implications for the treatment of heterozygous familial hypercholesterolemia. Am. J. Cardiol..

[10] Lambert K., Mullan J., Mansfield K. (2017). An integrative review of the methodology and findings regarding dietary adherence in end stage kidney disease. BMC Nephrol..

[11] Beerappa H., Chandrababu R. (2019). Adherence to dietary and fluid restrictions among patients undergoing hemodialysis: An observational study. Clin. Epidemiol. Glob. Health.

[12] Saran R., Bragg-Gresham J.L., Rayner H.C., Goodkin D.A., Keen M.L., Van Dijk P.C., Kurokawa K., Piera L., Saito A., Fukuhara S. (2003). Nonadherence in hemodialysis: Associations with mortality, hospitalization, and practice patterns in the DOPPS. Kidney Int..

[13] Zoccali C., Moissl U., Chazot C., Mallamaci F., Tripepi G., Arkossy O., Wabel P., Stuard S. (2017). Chronic Fluid Overload and Mortality in ESRD. J. Am. Soc. Nephrol..

[14] Tentori F., Blayney M.J., Albert J.M., Gillespie B.W., Kerr P.G., Bommer J., Young E.W., Akizawa T., Akiba T., Pisoni R.L. (2008). Mortality risk for dialysis patients with different levels of serum calcium, phosphorus, and PTH: The Dialysis Outcomes and Practice Patterns Study (DOPPS). Am. J. Kidney Dis..

[15] Wizemann V., Wabel P., Chamney P., Zaluska W., Moissl U., Rode C., Malecka-Masalska T., Marcelli D. (2009). The mortality risk of overhydration in haemodialysis patients. Nephrol. Dial. Transplant..

[16] Lambert K., Mansfield K., Mullan J. (2018). How do patients and carers make sense of renal dietary advice? A qualitative exploration. J. Ren. Care.

[17] Hollingdale R., Sutton D., Hart K. (2008). Facilitating dietary change in renal disease: Investigating patients’ perspectives. J. Ren. Care.

[18] Palmer S.C., Hanson C.S., Craig J.C., Strippoli G.F., Ruospo M., Campbell K., Johnson D.W., Tong A. (2015). Dietary and fluid restrictions in CKD: A thematic synthesis of patient views from qualitative studies. Am. J. Kidney Dis..

[19] Mellon L., Regan D., Curtis R. (2013). Factors influencing adherence among Irish haemodialysis patients. Patient Educ. Couns..

[20] Dageforde L.A., Cavanaugh K.L. (2013). Health literacy: Emerging evidence and applications in kidney disease care. Adv. Chronic Kidney Dis..

[21] World Health Organization (1998). Health Promotion Glossary. Health Promot. Int..

[22] Taylor D.M., Fraser S.D.S., Bradley J.A., Bradley C., Draper H., Metcalfe W., Oniscu G.C., Tomson C.R.V., Ravanan R., Roderick P.J. (2017). A Systematic Review of the Prevalence and Associations of Limited Health Literacy in CKD. Clin. J. Am. Soc. Nephrol..

[23] Green J.A., Mor M.K., Shields A.M., Sevick M.A., Palevsky P.M., Fine M.J., Arnold R.M., Weisbord S.D. (2011). Prevalence and demographic and clinical associations of health literacy in patients on maintenance hemodialysis. Clin. J. Am. Soc. Nephrol..

[24] Cavanaugh K.L., Wingard R.L., Hakim R.M., Eden S., Shintani A., Wallston K.A., Huizinga M.M., Elasy T.A., Rothman R.L., Ikizler T.A. (2010). Low health literacy associates with increased mortality in ESRD. J. Am. Soc. Nephrol..

[25] Taylor D.M., Fraser S., Dudley C., Oniscu G.C., Tomson C., Ravanan R., Roderick P., ATTOM investigators (2018). Health literacy and patient outcomes in chronic kidney disease: A systematic review. Nephrol. Dial. Transplant..

[26] Grubbs V., Gregorich S.E., Perez-Stable E.J., Hsu C.Y. (2009). Health literacy and access to kidney transplantation. Clin. J. Am. Soc. Nephrol..

[27] Fraser S.D., Roderick P.J., Casey M., Taal M.W., Yuen H.M., Nutbeam D. (2013). Prevalence and associations of limited health literacy in chronic kidney disease: A systematic review. Nephrol. Dial. Transplant..

[28] Green J.A., Mor M.K., Shields A.M., Sevick M.A., Arnold R.M., Palevsky P.M., Fine M.J., Weisbord S.D. (2013). Associations of health literacy with dialysis adherence and health resource utilization in patients receiving maintenance hemodialysis. Am. J. Kidney Dis..

[29] Tohme F., Mor M.K., Pena-Polanco J., Green J.A., Fine M.J., Palevsky P.M., Weisbord S.D. (2017). Predictors and outcomes of non-adherence in patients receiving maintenance hemodialysis. Int. Urol. Nephrol..

[30] Marcelli D., Kirchgessner J., Amato C., Steil H., Mitteregger A., Moscardò V., Carioni C., Orlandini G., Gatti E. (2001). EuCliD (European Clinical Database): A database comparing different realities. J. Nephrol..

[31] (2013). KDIGO 2012 Clinical Practice Guideline for the Evaluation and Management of Chronic Kidney Disease. Kidney Int. Suppl..

[32] Kolarcik P., Cepova E., Madarasova Geckova A., Elsworth G.R., Batterham R.W., Osborne R.H. (2017). Structural properties and psychometric improvements of the Health Literacy Questionnaire in a Slovak population. Int. J. Public Health.

[33] Osborne R.H., Batterham R.W., Elsworth G.R., Hawkins M., Buchbinder R. (2013). The grounded psychometric development and initial validation of the Health Literacy Questionnaire (HLQ). BMC Public Health.

[34] Dempster A.P., Laird N.M., Rubin D.B. (1997). Maximum Likelihood from Incomplete Data via the EM Algorithm. J. R. Stat. Soc. Ser. B.

[35] Beauchamp A., Buchbinder R., Dodson S., Batterham R.W., Elsworth G.R., McPhee C., Sparkes L., Hawkins M., Osborne R.H. (2015). Distribution of health literacy strengths and weaknesses across socio-demographic groups: A cross-sectional survey using the Health Literacy Questionnaire (HLQ). BMC Public Health.

[36] Adler N.E., Epel E.S., Castellazzo G., Ickovics J.R. (2000). Relationship of subjective and objective social status with psychological and physiological functioning: Preliminary data in healthy white women. Health Psychol..

[37] Demakakos P., Nazroo J., Breeze E., Marmot M. (2008). Socioeconomic status and health: The role of subjective social status. Soc. Sci. Med..

[38] Zou H., Chen Y., Fang W., Zhang Y., Fan X. (2016). The mediation effect of health literacy between subjective social status and depressive symptoms in patients with heart failure. J. Psychosom Res..

[39] Ranstam J. (2016). Multiple P-values and Bonferroni correction. Osteoarthritis Cartilage.

[40] IBM Corp. (2015). IBM SPSS Statistics for Windows, Version 23.0.

[41] Jain S., Fernando K., Hoover D.R. (2005). Health literacy skills of chronic hemodialysis patients. J. Am. Soc. Nephrol..

[42] Dodson S., Osicka T., Huang L., McMahon L.P., Roberts M.A. (2016). Multifaceted Assessment of Health Literacy in People Receiving Dialysis: Associations With Psychological Stress and Quality of Life. J. Health Commun..

[43] Stomer U., Goransson L., Wahl A., Urstad K. (2019). Health literacy in patients with chronic kidney disease stages 3–5. Nephrol. Dial. Transplant..

[44] Batterham R.W., Hawkins M., Collins P.A., Buchbinder R., Osborne R.H. (2016). Health literacy: Applying current concepts to improve health services and reduce health inequalities. Public Health.

[45] Jessup R.L., Osborne R.H., Beauchamp A., Bourne A., Buchbinder R. (2017). Health literacy of recently hospitalised patients: A cross-sectional survey using the Health Literacy Questionnaire (HLQ). BMC Health Serv. Res..

[46] Nutbeam D. (2000). Health Literacy as a public health goal: A challenge for contemporary health education and communication strategies info the 21st century. Health Promot. Int..

[47] Baker D.W. (2006). The meaning and the measure of health literacy. J. Gen. Intern. Med..

[48] Ishikawa H., Yano E. (2008). Patient health literacy and participation in the health-care process. Health Expect..

[49] Rüegg R., Abel T. (2019). The relationship between health literacy and health outcomes among male young adults: Exploring confounding effects using decomposition analysis. Int. J. Public Health.

